# No Evidence for Natural Selection on Endogenous Borna-Like Nucleoprotein Elements after the Divergence of Old World and New World Monkeys

**DOI:** 10.1371/journal.pone.0024403

**Published:** 2011-09-02

**Authors:** Yuki Kobayashi, Masayuki Horie, Keizo Tomonaga, Yoshiyuki Suzuki

**Affiliations:** 1 Graduate School of Natural Sciences, Nagoya City University, Nagoya, Aichi, Japan; 2 Research Institute for Microbial Diseases (BIKEN), Osaka University, Suita, Osaka, Japan; 3 Institute for Virus Research, Kyoto University, Sakyo-ku, Kyoto, Japan; Aarhus University, Denmark

## Abstract

Endogenous Borna-like nucleoprotein (EBLNs) elements were recently discovered as non-retroviral RNA virus elements derived from bornavirus in the genomes of various animals. Most of EBLNs appeared to be defective, but some of primate EBLN-1 to -4, which appeared to be originated from four independent integrations of bornavirus nucleoprotein (N) gene, have retained an open reading frame (ORF) for more than 40 million years. It was therefore possible that primate EBLNs have encoded functional proteins during evolution. To examine this possibility, natural selection operating on all ORFs of primate EBLN-1 to -4 was examined by comparing the rates of synonymous and nonsynonymous substitutions. The expected number of premature termination codons in EBLN-1 generated after the divergence of Old World and New World monkeys under the selective neutrality was also examined by the Monte Carlo simulation. As a result, natural selection was not identified for the entire region as well as parts of ORFs in the pairwise analysis of primate EBLN-1 to -4 and for any branch of the phylogenetic trees for EBLN-1 to -4 after the divergence of Old World and New World monkeys. Computer simulation also indicated that the absence of premature termination codon in the present-day EBLN-1 does not necessarily support the maintenance of function after the divergence of Old World and New World monkeys. These results suggest that EBLNs have not generally encoded functional proteins after the divergence of Old World and New World monkeys.

## Introduction

Endogenous Borna-like nucleoprotein (EBLN) elements were recently discovered as non-retroviral RNA virus elements in the genomes of various animals, including primates, rodents, chiropterans, afrotherians, and fishes [1], [2], [3]. EBLNs appeared to have been derived from genomic integrations of reverse-transcribed mRNAs for the nucleoprotein (N) gene of bornavirus, which is a non-segmented, single-stranded (negative sense) RNA virus [4], [5]. In primates, four copies of EBLNs (EBLN-1 to -4) were identified in the genomes of Old World and New World monkeys [1], [2], [3]. Each copy of EBLN-1 to -4 apparently started with the transcription start site of bornavirus N gene and ended with poly-A, and was flanked by the target site duplication, which was specific in length and sequence to each copy. These observations indicated that primate EBLN-1 to -4 were originated from four independent integrations of reverse-transcribed mRNA for bornavirus N gene by LINE before the divergence of Old World and New World monkeys (∼44.2 million years ago (MYA) in TIMETREE [6]) [1], [2].

Most of EBLNs appeared to be defective due to the existence of premature termination codons and frameshifts [2]. However, EBLN-1 of human (367 codons), chimpanzee (368 codons), and gorilla (368 codons) encoded an open reading frame (ORF), which was almost equivalent in length to the N gene of bornavirus (371–374 codons), indicating that the ORF has been maintained for more than 40 million years along the evolutionary lineage leading to these organisms. This phenomenon was considered unlikely to be observed in the absence of purifying selection on EBLN-1 [1], [3]. In addition, human EBLN-1 to -4 were identified to be expressed in various tissues, and human EBLN-2 was found to interact with other proteins [1], [2], [7]. These observations raised a possibility that primate EBLNs have been functional in the host.

If primate EBLNs have encoded functional proteins, natural selection, either positive or negative, should have operated on them during evolution of primates. Natural selection operating at the amino acid sequence level may be detected by comparing the rate of nonsynonymous substitution (*d*_N_) with that of synonymous substitution (*d*_S_), where the relationships *d*_N_>*d*_S_, *d*_N_<*d*_S_, and *d*_N_ = *d*_S_ indicate positive, negative, and no selection, respectively [8]. The purpose of the present study was to examine the functionality of EBLNs during primate evolution by identifying natural selection from the comparison of *d*_N_ and *d*_S_.

## Results

### ORFs in primate EBLN-1 to -4

**Figure 1:**
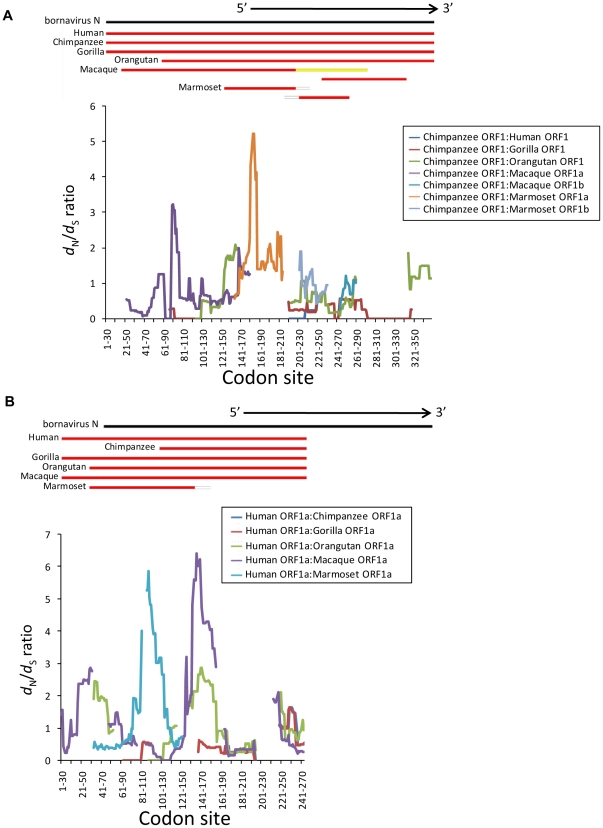
Sliding-window analysis of ORF1s in EBLN-1 and -2. (A) ORF1 of EBLN-1 and (B) ORF1a of EBLN-2. The *d*_N_/*d*_S_ ratio is plotted as the colored lines along ORFs. Disconnected lines indicate the regions where no synonymous substitution was observed.

Single orthologous regions of EBLN-1 to -4 were identified in the genomes of all primates analyzed in the present study (human, chimpanzee, gorilla, orangutan, macaque, and marmoset), except for the orthologous region of EBLN-4 in the marmoset genome, which has duplicated multiple times (Figures S1 and S2, Table S1) [3]. Macaque EBLN-1 and marmoset EBLN-4 contained sequences that were derived from *Alu* repeat elements (Figure 1), which were characterized as *Alu*MacYa3, belonging to the macaque-specific *Alu* subfamily [9], and as *Alu*Sp or *Alu*2, respectively.

In order to investigate the functionality of primate EBLNs during evolution, a total of 100 ORFs with >50 codons was identified in the orthologous regions of EBLN-1 to -4 in the primate genomes (Figure S1, Table S1). Most of these ORFs were not shared among the primates examined, because they were truncated due to premature termination codons in some species (Figure S1). However, ORF1s of EBLN-1 in human (367 codons), chimpanzee (368 codons), and gorilla (368 codons) were almost equivalent in length to bornavirus N gene (371–374 codons). In addition, the size of ORF1a of EBLN-2 in gorilla and macaque was equal to that in human (273 codons), which is known to interact with other proteins [7]. ORF1a of macaque EBLN-1 contained the sequence derived from *Alu*MacYa3 [9]. Parts of ORFs in marmoset EBLN-4 were also derived from transposable elements (*Alu*Sp or *Alu*2 for ORF2 of contig1733, ORF–1 of contig1733 and 1917, and ORF–2a of contig1129 and 6507, and *Alu2* and LIME1 for ORF–2 of contig1129, 1952, 5225, and 6507).

### Natural selection operating on ORFs in primate EBLN-1 to -4

In the above analysis, ORF1 of EBLN-1 and ORF1a of EBLN-2 were found to be relatively long in some primates. However, when the entire region of ORF1 of EBLN-1 was compared between primates, the *d*_N_/*d*_S_ ratio ranged from 0.65 to 2.68, and no signature of natural selection was identified (*Z*-test: *p*>0.06) (Table 1). Similarly, no selection was detected for ORF1a of EBLN-2 (*Z*-test: *p*>0.1) as well as all other ORFs of EBLN-1 to -4 after the correction for multiple testing (*Z*-test: *p*>0.03) (Tables 1 and S2). In the window analysis, no common pattern was observed for the fluctuation of *d*_N_/*d*_S_ ratio along linear sequences of ORFs in EBLN-1 to -4 between pairs of primates (Figures 1 and S3). Natural selection was not detected at any window between any pair of primates (*Z*-test: *p*>0.35).

**Table 1:** *d*_N_/*d*_S_ ratios between pairs of orthologous sequences of ORF1 in EBLN-1 and -2.

EBLN	ORF pairs	codons^a^	*d*_N_	*d*_S_	*d*_N_ */d*_S_	*p* b
EBLN-1	Human ORF1:Chimpanzee ORF1	365	0.0153	0.0080	1.9296	0.24
	Human ORF1:Macaque ORF1a	194	0.0521	0.0796	0.6538	0.33
	Human ORF1:Gorilla ORF1	367	0.0161	0.0141	1.1418	0.79
	Human ORF1:Orangutan ORF1	303	0.0411	0.0377	1.0888	0.82
	Chimpanzee ORF1:Macaque ORF1a	195	0.0561	0.0720	0.7791	0.59
	Chimpanzee ORF1:Gorilla ORF1	367	0.0171	0.0220	0.7773	0.63
	Chimpanzee ORF1:Orangutan ORF1	305	0.0439	0.0424	1.0342	0.93
	Macaque ORF1a:Gorilla ORF1	195	0.0520	0.0705	0.7376	0.52
	Macaque ORF1a:Orangutan ORF1	150	0.0482	0.0514	0.7845	0.91
	Gorilla ORF1:Orangutan ORF1	305	0.0366	0.0545	0.6716	0.35
	Human ORF1:Macaque ORF1b	94	0.0824	0.0307	2.6845	0.06
	Human ORF1:Marmoset ORF1a	78	0.0933	0.1338	0.6974	0.51
	Human ORF1:Marmoset ORF1b	59	0.1526	0.0888	1.7178	0.29
	Chimpanzee ORF1:Macaque ORF1b	94	0.0704	0.0407	1.7308	0.37
	Chimpanzee ORF1:Marmoset ORF1a	80	0.1005	0.1292	0.7780	0.64
	Chimpanzee ORF1:Marmoset ORF1b	59	0.1299	0.1069	1.2152	0.75
	Macaque ORF1a:Marmoset ORF1a	80	0.1174	0.1048	1.1198	0.81
	Macaque ORF1b:Gorilla ORF1	94	0.0643	0.0835	0.7701	0.65
	Macaque ORF1b:Orangutan ORF1	94	0.0419	0.0306	1.3690	0.67
	Gorilla ORF1:Marmoset ORF1a	80	0.1119	0.1274	0.8789	0.79
	Gorilla ORF1:Marmoset ORF1b	59	0.1270	0.1060	1.1981	0.76
	Orangutan ORF1:Marmoset ORF1a	80	0.1005	0.1022	0.9839	0.97
	Orangutan ORF1:Marmoset ORF1b	59	0.1077	0.0705	1.5282	0.51
EBLN-2	Human ORF1a:Macaque ORF1a	272	0.0627	0.0746	0.8400	0.62
	Human ORF1a:Orangutan ORF1a	241	0.0449	0.0407	1.1040	0.83
	Human ORF1a:Chimpanzee ORF1a	163	0.0206	0.0204	1.0093	0.99
	Human ORF1a:Gorilla ORF1a	272	0.0225	0.0160	1.4063	0.58
	Human ORF1a:Marmoset ORF1	119	0.1245	0.1868	0.6662	0.28
	Macaque ORF1a:Orangutan ORF1a	241	0.0535	0.0733	0.7305	0.45
	Macaque ORF1a:Chimpanzee ORF1a	163	0.0458	0.0809	0.5657	0.27
	Macaque ORF1a:Gorilla ORF1a	272	0.0489	0.0640	0.7641	0.46
	Macaque ORF1a:Marmoset ORF1	119	0.1141	0.2278	0.5010	0.10
	Orangutan ORF1a:Chimpanzee ORF1a	163	0.0402	0.0323	1.2437	0.70
	Orangutan ORF1a:Gorilla ORF1a	241	0.0365	0.0301	1.2126	0.70
	Orangutan ORF1a:Marmoset ORF1	119	0.1293	0.2195	0.5889	0.14
	Chimpanzee ORF1a:Gorilla ORF1a	163	0.0080	NA^c^	NA^d^	NA^d^
	Marmoset ORF1:Gorilla ORF1a	119	0.1080	0.2058	0.5248	0.10
	Human ORF1b:Chimpanzee ORF1b	72	0.0071	0.0288	0.2455	0.33
	Human ORF1b:Gorilla ORF1b	76	0.0069	0.0124	0.5588	0.71
	Chimpanzee ORF1b:Gorilla ORF1b	55	0.0000	0.0186	0.0000	0.37

a The number of codons used for the estimation of *d*_N_/*d*_S_ ratio.

b *p*-value obtained by the *Z*-test.

c Not applicable because the number of synonymous sites was 0.

d Not applicable because the *d*_S_ was NA.

**Figure 2:**
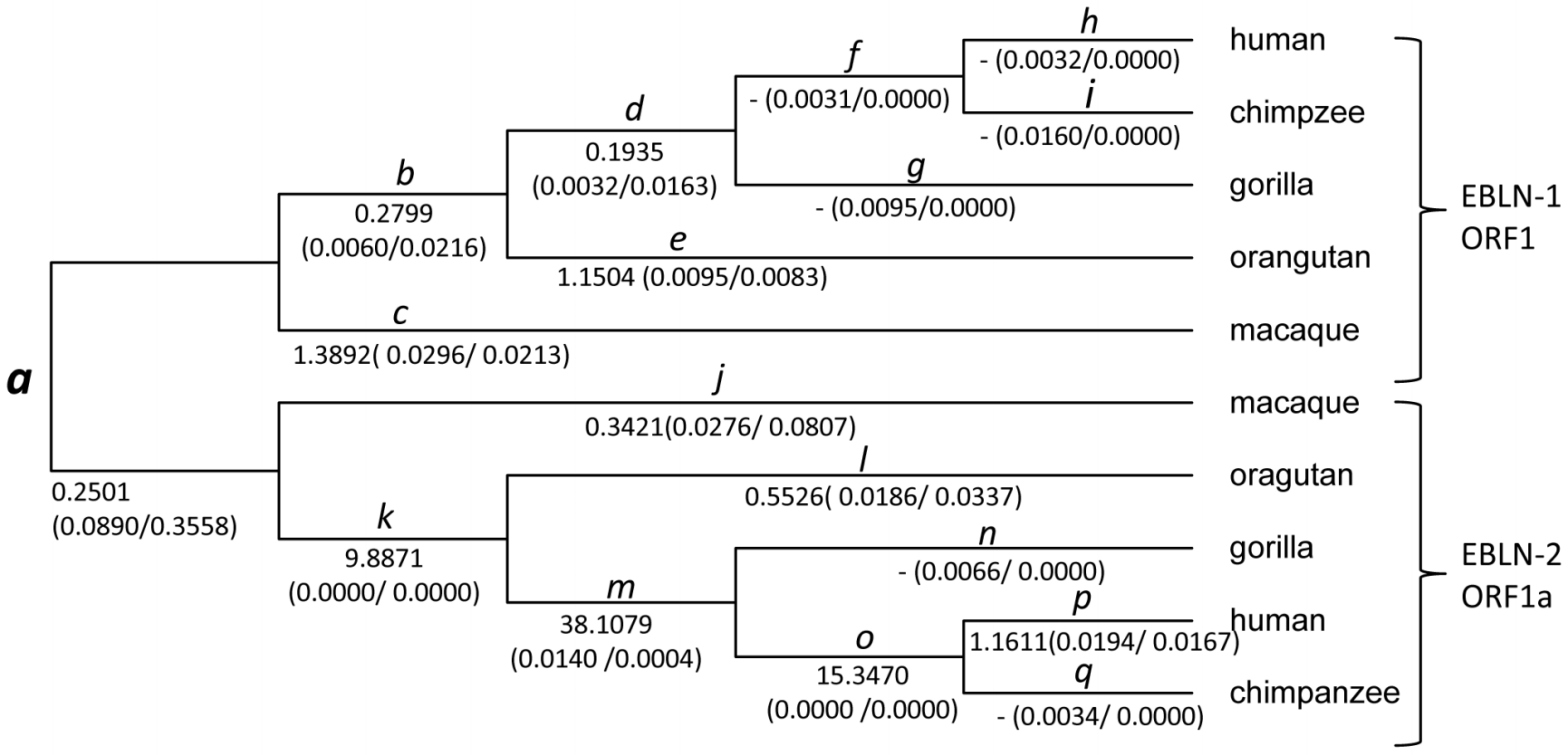
The *d*_N_/*d*_S_ ratios estimated at each branch of the phylogenetic tree for EBLN-1 and -2. The values under the branches show the *d*_N_/*d*_S_ ratio (*d*_N_ value/*d*_S_ value), which was estimated under the selection model. The branch where negative selection was detected is labeled with the bold letter.

When natural selection was examined at each branch of the phylogenetic trees for ORFs of EBLN-1 to -4, negative selection was detected at the basal branch of the phylogenetic tree for ORF1 of EBLN-1 and ORF1a of EBLN-2 after the correction for multiple testing (branch *a* in Figure 2; *d*_N_/*d*_S_ ratio  = 0.25, likelihood ratio test (LRT): *p* = 4.52×10^−6^) (Table S3). It should be noted that this branch reflects the evolution of not only EBLN-1 and -2 but also bornavirus N gene before integration, because two independent integrations of bornavirus N gene appears to have taken place to give rise to EBLN-1 and -2 on this branch, as discussed above. No selection was detected at any other branches of the phylogenetic trees reflecting the evolution of EBLN-1 to -4 after the correction for multiple testing (LRT: *p*>1.34×10^−3^) (Figures 2 and S4, Table S3).

### Probability of maintaining ORF1 of EBLN-1 after the divergence of Old World and New World monkeys under the selective neutrality

Two studies have argued for the possibility that purifying selection has operated on ORF1 of EBLN-1 after the divergence of Old World and New World monkeys [1], [3]. Both arguments were based on the observation that the premature termination codon was absent in ORF1s of present-day EBLN-1 for human, chimpanzee, and gorilla, which was considered to be unexpected under the selective neutrality [1], [3].

In the first study [1], the generation rate of termination codon was computed theoretically based on the assumption that the rate of nucleotide substitution was 1.2×10^−9^ per site per year under the selective neutrality. It was claimed that there is a 12% probability that a random codon change will produce a termination codon in one mutational step when the rates of all nucleotide substitutions are the same. The generation rate of termination codon was estimated to be 1 per 2,310 codons per million years [1]. Therefore, if we assume that the divergence time of Old World and New World monkeys was 44.2 MYA or 54.1 MYA and the ancestral EBLN-1 consisted of 371 codons, 7.08 or 8.67 premature termination codons were expected to be observed in the present-day EBLN-1, respectively, which were much greater than zero [1].

In the second study [3], the Monte Carlo simulation was conducted for the evolution of EBLN-1 after the divergence of Old World and New World monkeys under the selective neutrality. It was assumed that the divergence time of Old World and New World monkeys was 54.1 MYA and the rate of nucleotide substitution under the selective neutrality was 2.2×10^−9^ per site per year. When the consensus sequence of EBLN-1 was evolved according to this evolutionary scenario, the number of premature termination codons in the present-day EBLN-1 was 15.57 on average and the probability of observing zero premature termination codon was *p*<0.00001 [3].

However, there appeared some problems in these studies [1], [3]. First, it is well-known that the proportion of nucleotide substitution producing termination codons under the assumptions of equal rates for all nucleotide substitutions and equal frequencies for all codons is 4% [10], which is one-third of the value (12%) assumed in [1]. Therefore, the expected number of premature termination codons in the present-day EBLN-1 may not be 7.08 or 8.67 but 2.36 or 2.89, which is not very much different from zero. In addition, in [3], the expected number of nucleotide substitutions occurring in 371 codons (1,113 nt) during 54.1 million years under the rate of 2.2×10^−9^ per site per year is 132.11. If we assume that the probability for a nucleotide substitution to produce a termination codon is 4%, the expected number of premature termination codons in the present-day EBLN-1 is 5.3 (132.11×0.04), which is much smaller than 15.57 predicted by the simulation [3].

Second, the divergence time of Old World and New World monkeys (54.1 MYA) and the rate of nucleotide substitution in primates (2.2×10^−9^ per site per year) assumed in [3] may not be appropriate. In fact, the divergence time of Old World and New World monkeys has been estimated to be 44.2 MYA in TIMETREE [6]. In addition, the evolutionary rate of 2.2×10^−9^ per site per year appears to have been reported as the average rate for mammals [11], which is higher than that estimated from the analysis of primate genomes evolving under the selective neutrality (0.99–1.5×10^−9^ per site per year) [12].

**Figure 3:**
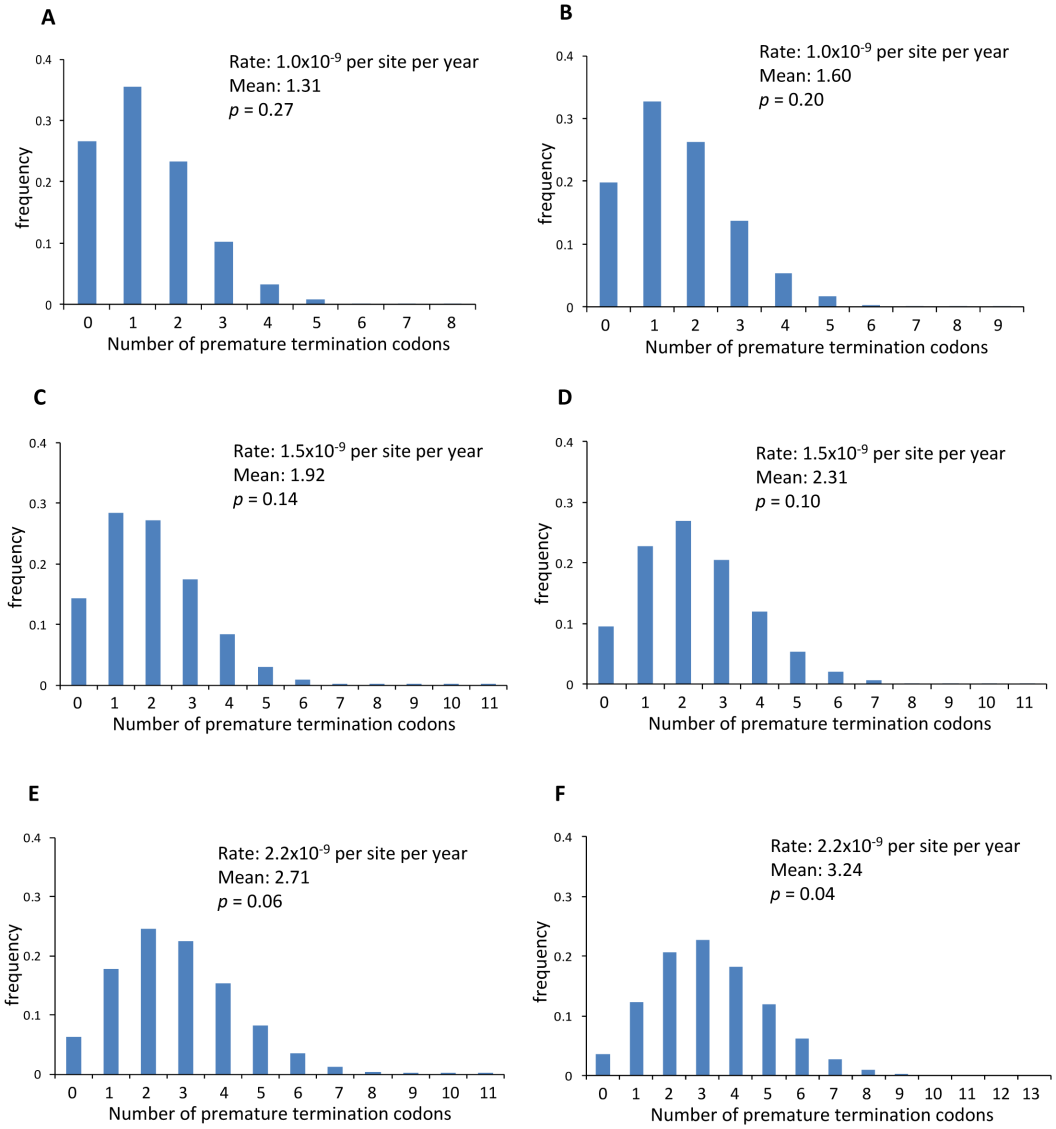
The distribution of the number of premature termination codons in the computer simulation. The consensus primate EBLN-1 sequence was evolved for (A, C, E) 44.2 million years and (B, D, F) 54.1 million years with the rate of (A, B) 1.0, (C, D) 1.5, and (E, F) 2.2×10^−9^ per site per year. Mean indicates the average number of premature termination codons, and *p* indicates the probability of observing zero premature termination codon in the simulated sequence.

For these reasons, we performed the Monte Carlo simulation for the evolution of EBLN-1 after the divergence of Old World and New World monkeys, similarly to that conducted in [3], under various conditions. When the consensus sequence of EBLN-1 (kindly provided by Dr. Robert J. Gifford) was evolved for 54.1 million years with the rate of 2.2×10^−9^ per site per year, which mimics the simulation in [3], the average number of premature termination codons was 3.24 (Figure 3F), which was significantly smaller than that (15.57) obtained in [3]. In addition, the probability of observing zero premature termination codon was 0.04, which was significantly greater than that (*p*<0.00001) obtained in [3]. Although the reason for this discrepancy was unclear, it may be noted that a somewhat similar result to [3] (average number of premature termination codons  = 13.54 and *p*<0.00001) was observed when the divergence time or the rate was assumed to be ten times greater than that used above (541 MYA instead of 54.1 MYA or 22×10^−9^ per site per year instead of 2.2×10^−9^ per site per year) (Figure S5).

In the above simulation, the probability of observing zero premature termination codon was smaller than 0.05 (*p* = 0.04) under the assumptions of the divergence time of 54.1 MYA and the rate of 2.2×10^−9^. Interestingly, however, the probability increased when the assumed divergence time was changed from 54.1 MYA (*p* = 0.04) to a more realistic value of 44.2 MYA (*p* = 0.06) and the assumed rate was changed from 2.2×10^−9^ (*p* = 0.06) to more realistic values of 1.5×10^−9^ (*p* = 0.14) and 1.0×10^−9^ (*p* = 0.27) (Figure 3A–3E). Similar results were obtained when the inferred ancestral sequence and the present-day bornavirus N sequence were evolved in the simulation (Figures S6 and S7). These observations indicate that the absence of premature termination codon in the present-day EBLN-1 does not necessarily support the maintenance of function after the divergence of Old World and New World monkeys.

## Discussion

Natural selection was not detected for the entire region or parts of the ORFs of EBLN-1 to -4 between any pair of primates and for any branch of the phylogenetic trees for EBLN-1 to -4 after the divergence of Old World and New World monkey, suggesting that primate EBLN-1 to -4 have not encoded functional proteins during this period. These results conflicted with those in the previous studies, which suggested that purifying selection has operated on EBLN-1 to maintain ORF1 during this period [1], [3]. However, there appeared some problems in the previous studies, as discussed above. When the divergence time of Old World and New World monkeys and the neutral substitution rate assumed in the previous study [3] were changed to more realistic values, the probability of observing zero premature termination codon was found to be *p*>0.06 in the Monte Carlo simulation. Therefore, the absence of premature termination codon in ORF1s of the present-day EBLN-1 does not necessarily support the maintenance of function after the divergence of Old World and New World monkeys. The truncation of ORF1 due to premature termination codons in orangutan EBLN-1 and the insertion of an *Alu* element in macaque EBLN-1 also support the absence of functional constraint on EBLN-1.

It should be noted, however, that negative selection was detected at the basal branch of the phylogenetic tree for ORF1 of EBLN-1 and ORF1a of EBLN-2. This branch appeared to represent the evolution of bornavirus N gene before integration as well as EBLN-1 and -2 before the divergence of Old World and New World monkeys. Since the *d*_N_/*d*_S_ ratio for bornavirus N gene is generally smaller than unity (data not shown), negative selection identified for the basal branch of EBLN-1 and -2 likely reflects the functional constraint on bornavirus N gene before integration. However, it might be possible that negative selection has also operated on EBLN-1 and -2 before the divergence of Old World and New World monkeys. This is because expression of bornavirus N protein is known to inhibit replication of bornavirus in rats and mice [13], [14], and therefore EBLN-1 and -2, if expressed as is the case for present-day EBLN-1 and -2 in humans, might have conferred selective advantages to ancient primates by preventing bornavirus infection. In this regard, it is interesting to note that EBLNs have been observed in the genomes of primates, rodents, chiropterans, opossums, afrotherians, and fishes [1], [2], where prevalence of bornavirus has not been reported. In contrast, birds, cattle, sheep, and horse, which do not harbor EBLNs, are known as the natural hosts of bornavirus [4], [5], [15]. It might be speculated that as EBLNs protected ancient primates from infection by primate bornavirus, this virus may have changed the host preference or become extinct, which may have promoted the relaxation of functional constraint on EBLNs after the divergence of Old World and New World monkeys.

In conclusion, no evidence for natural selection was identified for EBLN-1 to -4 after the divergence of Old World and New World monkeys. In the present study, we mainly focused on the functionality of EBLNs as proteins. However, considering that mRNA expression of EBLNs has been detected in various tissues [1], [2], it is tempting to speculate that EBLNs might have been functional as RNAs. In addition, interactions identified between human EBLN-2 and cellular proteins [7] point to a possibility that human EBLN-2 might have been acquiring a new function. Further studies are needed to fully understand the functional significance of EBLNs integrated into the host genome.

## Materials and Methods

### Identification of ORFs in primate EBLN-1 to -4

The nucleotide sequences of EBLN-1 to -4, which were reported in Horie *et al*. (2010) [2], as well as their flanking 1,000 nt up-stream and down-stream each in primate genomes (human, chimpanzee, gorilla, orangutan, macaque, and marmoset) were retrieved from the Ensemble Genome Browser. To examine the entire coding capacity of EBLN-1 to -4, all ORFs with >50 codons overlapping with the original EBLN-1 to -4 were extracted. The ORFs whose first codon shared the reading frame with bornavirus N gene (isolate H1499; Accession no. AY374520) were named ORF1. The ORFs encoded on the same strand as ORF1 were named ORF2 and ORF3, when position 1 of the first codon corresponded to positions 2 and 3 of codons in bornavirus N gene, respectively. The ORFs encoded on the opposite strand of ORF1 were named ORF–1, ORF–2, and ORF–3, when position 1 of the first codon was complementary to positions 1, 2, and 3 of codons in bornavirus N gene, respectively. Some of ORF1, ORF2, ORF3, ORF–1, ORF–2, and ORF–3 were named ORF1a, ORF1b, and so on, when they were partial. Undetermined nucleotides in gorilla, orangutan, and marmoset were treated as gaps in the analysis. Transposons found in the ORFs were classified using Repbase [16].

### Phylogenetic analysis of primate EBLN-1 to -4

Multiple alignment of nucleotide sequences for primate EBLN-1 to -4 was made by using the computer program Clustal W [17]. The general time reversible model (GTR) with the gamma distribution for the rate heterogeneity among sites (G) was selected as the optimum model of nucleotide substitution for primate EBLN-1 to -4 by MODELTEST [18] with PAUP (ver. 4.0). Phylogenetic tree for EBLN-1 to -4 was constructed by the neighbor-joining (NJ) [19], maximum likelihood (ML) [20], and Bayesian methods with GTR+G by using PAUP, PhyML (ver. 3.0) [21], and MrBayes (ver. 3.1) [22], respectively. The reliability for the NJ and ML trees was assessed using the bootstrap probability with 1,000 and 100 re-samplings, respectively. To construct the Bayesian tree, the Markov chain Monte Carlo chains were run for 1,000,000 generations with a burn-in of first 25,000 generations, and the phylogenetic tree was sampled every 1,000 generations. The credibility of the interior branch was assessed as the posterior probability.

### Statistical analysis of natural selection operating on ORFs of primate EBLN-1 to -4

For each of EBLN-1 to -4, multiple alignments of nucleotide sequences for ORFs were made by using Clustal W. Natural selection operating over the entire ORFs was inferred by computing the *d*_N_/*d*_S_ ratio between pairs of orthologous sequences using the Pamilo-Bianchi-Li method [23], [24] with the pairwise deletion option in MEGA. The standard errors of *d*_N_ and *d*_S_ were estimated using the bootstrap method with 1,000 re-samplings, and the null hypothesis of no selection (*d*_N_ = *d*_S_) was tested by the *Z*-test [10]. Bonferroni correction was applied to account for multiple testing, where the significance level for individual tests was modified by considering the number of independent tests with the family-wise significance level set at *p* = 0.05. Natural selection operating on particular regions of ORFs was examined by the sliding window analysis using CRANN [25]. The *d*_N_/*d*_S_ ratio between orthologous sequences was estimated using the Pamilo-Bianchi-Li method for each window (window size  = 30 codons, step size  = 1 codon). The null hypothesis of no selection (*d*_N_ = *d*_S_) was tested by the *Z*-test.

Natural selection was also examined at each branch of the phylogenetic trees for ORFs of EBLN-1 to -4. The topology of the species tree was used in the analysis, because EBLN-1 to -4 of all primates analyzed in the present study were considered to be otrthologous [2] and the topology within each cluster of EBLN-1 to -4 in the NJ, ML, and Bayesian trees was almost the same as that for the species tree of primates (Figure S2). The *d*_N_/*d*_S_ ratio was estimated for each branch by the maximum likelihood method using the codon substitution model in PAML (version 4.0) [26]. The equilibrium codon frequencies were treated as free parameters, and the *d*_N_/*d*_S_ ratio was estimated under the free-ratio (selection) model and the branch specific (null) model. In the selection model, the *d*_N_/*d*_S_ ratio was allowed to vary among branches, whereas in the null model, the *d*_N_/*d*_S_ ratio was fixed to be 1 at specified branches. Since different results can be obtained depending on the initial *d*_N_/*d*_S_ ratio in PAML [27], 0.4, 1, and 3.14 (and 15 in some cases) were used as the initial *d*_N_/*d*_S_ ratio, and the results with the highest likelihood values were adopted as the final results.

The null hypothesis of no selection (*d*_N_/*d*_S_ ratio  = 1) for the specified branch was tested by the LRT, where twice the difference in the log-likelihood value was assumed to follow a χ^2^ distribution with the degree of freedom equal to the difference in the number of parameters estimated in the null and selection models. Since the LRT was conducted for a total of 108 branches in the phylogenetic trees for EBLN-1 to -4, the significance level for individual tests was set at *p* = 0.00046, which corresponded to the family-wise significance level of *p* = 0.05, using the Bonferroni correction for multiple testing.

### Simulation

The probability that primate EBLN-1 has maintained ORF1 under the selective neutrality (no selection) after the divergence of Old World and New World monkeys was obtained by simulating the evolution of primate EBLN-1 using Seq-Gen [28]. The ancestral sequence of primate EBLN-1 (1,104nt) was inferred from the sequences of EBLN-1s for human, chimpanzee, gorilla, orangutan, and macaque using the Bayesian method implemented in PAML. Macaque EBLN-1, from which the *Alu* element was removed, was used as the outgroup to determine the position of the root for other sequences, where the ancestral sequence was inferred. The sequences of inferred ancestral primate EBLN-1 (1,104nt), consensus primate EBLN-1 (1,104nt) reported previously [3], and bornavirus N gene (1,113nt) (isolate H1499; Accession no.: AY374520) were evolved after removing termination codons with three evolutionary rates (1.0×10^−9^, 1.5×10^−9^, and 2.2×10^−9^ per site per year [3], [11], [12]) and two divergence times of Old World and New World monkeys (44.2 MYA and 54.1 MYA [3], [6]). The transition/transversion rate ratio was assumed to be 4. The number of premature termination codons in the simulated sequence was counted for 100,000 iterations.

## Supporting Information

Figure S1 **Identification of ORFs in EBLN-1 to -4.** (A) EBLN-1, (B) EBLN-2, (C) EBLN-3, and (D) EBLN-4. Each ORF is located to show the positional correspondence to the amino acid sequence of bornavirus N gene. The black, red, blue, orange, and yellow bars indicate the sequences of bornavirus N gene, ORF1 or ORF–1, ORF2 or ORF–2, ORF3 or ORF–3, and transposon, respectively. The white bars indicate the regions with frameshifts. The digits at the ends of bars denote the codon numbers in ORFs.
(PPTX)

Figure S2 **ML tree of primate EBLN-1 to -4.** The credibility values are attached to the interior branches that are supported by all three methods with >70% bootstrap or posterior probability (NJ bootstrap probability/ML bootstrap probability/Bayesian posterior probability). The scale bar indicates the number of nucleotide substitutions per site.
(PPTX)

Figure S3 **Sliding-window analysis of the** ***d*** **_N_/** ***d*** **_S_ ratio.** (A) ORF2 of EBLN-1, (B) ORF–2 of EBLN-1, (C) ORF1 of EBLN-3, (D) ORF–1 of EBLN-3, (E) ORF1 of EBLN-4, and (F) ORF–1 to –3 of EBLN-4. The *d*_N_/*d*_S_ ratio is plotted as the colored lines along ORFs. Disconnected lines show the regions where no synonymous substitution was observed. The sliding-window analysis between pairs of ORF1 in marmoset EBLN-4 (contig1858, 1733, 5229, and 1952) was not conducted because the nucleotide sequences of contig1858 and 1733 were completely identical, and no synonymous substitution was observed in the comparison of contig1858, 5225, and 1952.
(PPTX)

Figure S4 **The** ***d*** **_N_/** ***d*** **_S_ ratio estimated for each branch of the phylogenetic trees.** The phylogenetic trees for (A) ORF1 of EBLN-1 and ORF1b of EBLN-2, (B) ORF2 of EBLN-2, (C) ORF–2 of EBLN-1 and EBLN-2, (D) ORF–1 of EBLN-2, (E) ORF1a of EBLN-3, (F) ORF1b of EBLN-3, (G) ORF–1a of EBLN-3, (H) ORF–1b of EBLN-3, (I) ORF1b of EBLN-4, (J) ORF1 of EBLN-3 and EBLN-4, (K) ORF–1 of EBLN-4, (L) ORF–1 of EBLN-4, (M) ORF–2 of EBLN-4, and (N) ORF–3 of EBLN-4. The values under the branches show the *d*_N_/*d*_S_ ratio (*d*_N_ value/*d*_S_ value), which was estimated under the selection model.
(PPTX)

Figure S5 **The distribution of the number of premature termination codons in the computer simulation.** The consensus primate EBLN-1 sequence was evolved for 541 million years with the rate of 2.2×10^−9^ per site per year (or for 54.1 million years with the rate of 22×10^−9^ per site per year). Mean indicates the average number of premature termination codons, and *p* indicates the probability of observing zero premature termination codon in the simulated sequence.
(PPTX)

Figure S6 **The distribution of the number of premature termination codons in the computer simulation.** The inferred ancestral sequence was evolved for (A, C, E) 44.2 million years and (B, D, F) 54.1 million years with the rate of (A, B) 1.0, (C, D) 1.5, and (E, F) 2.2×10^−9^ per site per year. Mean indicates the average number of premature termination codons, and *p* indicates the probability of observing zero premature termination codon in the simulated sequence.
(PPTX)

Figure S7 **The distribution of the number of premature termination codons in the computer simulation.** The bornavirus N sequence was evolved for (A, C, E) 44.2 million years and (B, D, F) 54.1 million years with the rate of (A, B) 1.0, (C, D) 1.5, and (E, F) 2.2×10^−9^ per site per year. Mean indicates the average number of premature termination codons, and *p* indicates the probability of observing zero premature termination codon in the simulated sequence.
(PPTX)

Table S1 **Genome positions of ORFs in EBLN-1 to -4.** ^a^Genome position corresponding to the primate genomes in the Ensemble Genome Browser. ^b^Sequences containing undetermined nucleotides. ^c^ORF with >50 codons was not identified.
(XLSX)

Table S2 ***d*** **_N_/** ***d*** **_S_ ratios between pairs of orthologous sequences of ORFs in EBLN-1 to -4.** ^a^The number of codons used for the estimation of *d*_N_/*d*_S_ ratio. ^b^ *p*-value obtained by the *Z*-test. ^c^Not applicable because the number of synonymous sites was 0. ^d^Not applicable because the *d*_S_ was 0 or NA. ^e^Not applicable because the *d*_S_ was NA.
(XLSX)

Table S3 ***d*** **_N_/** ***d*** **_S_ ratio at each branch of the phylogenetic trees for EBLN-1 to -4.** ^a^The number of codons used for the estimation of *d*_N_/*d*_S_ ratio. ^b^Branch name of the phylogenetic tree. ^c^ *d*_N_/*d*_S_ ratio estimated in the present study. ^d^Likelihood value estimated under the null model. ^e^Likelihood value estimated under the selection model. ^f^ *p*-value obtained by the LRT. ^g^Not applicable because the *d*_S_ was 0. ^h^Not applicable because InL1 was greater than InL2.
(XLSX)
